# Suicidal Ideation and Migration Aspirations among Youth in Central Mexico

**DOI:** 10.5539/gjhs.v6n1p92

**Published:** 2013-10-16

**Authors:** Steven Hoffman

**Affiliations:** 1 Department of Social Work, University of Texas at San Antonio, San Antonio, Texas, USA

**Keywords:** migration, suicide, youth, Mexico, suicidal ideation

## Abstract

Over the past 100 years the state of Guanajuato has consistently been one of the highest migrant sending states in Mexico. Youth living in high migratory states such as Guanajuato are heavily influenced by the expectation that they will travel to the US, and research has shown that those who do not migrate may be looked down upon by members of their community. This secondary analysis looks at the connection between suicidal ideation and migration aspirations among a group of adolescents living in Guanajuato, Mexico. Data were originally collected in 2007 as part of a comprehensive health survey of youth attending an alternative high schooling program. Regression analyses show that suicidal ideation predicts intentions to migrate among both males and females, while other factors differentially influence the adolescents by gender. The results indicate that suicidal ideation may be associated with migration aspirations among Mexican youth living in high migratory communities. Study limitations and implications are discussed.

## 1. Background

### 1.1 Suicide

Suicide is a leading cause of death throughout the world (Legleye et al., 2009), and can be a source of various problems at both the micro (individual) and macro (societal) levels. At the micro level, suicide survivors (i.e., those who have lost a loved one to suicide) face psychological, emotional, and familial hardships that negatively impact their quality of life. Common hardships include depression, guilt, anxiety, and general distress (Mcmenamy, Jordan, & Mitchell, 2008). On a macro level, the impact of suicide on a country’s economy can be substantial. For example, it has been estimated that suicide in the US leads to over $111 billion lost each year (Miller, Covington, & Jensen, 1999). The devastating individual and societal effects of suicide have led governments throughout the world to develop national strategies to better understand and reduce this widespread problem (e.g., US Department of Health and Human Services, 2001).

While many nations grapple with the challenges of high suicide mortality, Mexico has experienced a comparatively low suicide rate of 3.72/100,000 (Hernandez, A. Leenaars, Sanchez, & L. Leenaars, 2009; Puentes-Rosas, López-Nieto, & Martínez-Monroy, 2004). In fact, in a study that compared the suicide rates of 50 countries throughout the world, the suicide rate in Mexico ranked as the 4^th^ lowest (Helliwell, 2007). While international comparisons of suicide rates may lead to the belief that suicide in Mexico is not a pressing problem, a closer investigation into the country’s historical suicide rates shows a concerning trend. Between 1970 and 1994 the suicide rate in Mexico rose from 1.13/100,000 to 2.89/100,000 (Santos-Preciado et al., 2003). Furthermore, in 1994, the rate increased an unprecedented 156% (Santos-Preciado et al., 2003), and from 1970 to 2007 it has increased 275% (Borges, Orozco, Benjet, & Medina-Mora, 2010). When compared to 28 other countries between 1990 and 2000, Mexico’s suicide rate increase was one of the highest (Borges et al., 2008). Among the working age group since 2001, suicide is the 9^th^ most common cause of death (Hernandez et al., 2009). Of particular concern were Mexican adolescent suicide trends between 1990 and 2001, during which time the suicide rate for youth ages 11-19 increased from 0.8/100,000 - 2.27/100,000 among girls, and 2.6/100,000 - 4.5/100,000 among boys (Puentes-Rosas et al., 2004). In the state of Guanajuato, even more concerning than the growing suicide rate is the percentage of suicides being carried out by youth. Between 1997 and 2001, 65% of all suicides in Guanajuato were committed by adolescents (Chiapas, 2008). Unfortunately, theories seeking to explain this astonishingly high percentage of adolescent suicides in Guanajuato are not presently found in the research literature.

### 1.2 Migration

Migration, especially Mexican migration to the US, has been studied for years in an attempt to understand the motivating factors behind the decision to leave one’s country of origin. A review of this literature shows that some of the reasons behind the decision to migrate are: 1) large-scale violence (Martin & Widgren, 2002); 2) domestic violence (Klevens, 2007); 3) the prospect for economic advancement (Alba, Massey, & Rumbaut, 1999); and 4) family reunification (Durand, Nolan, & Massey, 2003). One motivating factor that is especially influential among individuals living in central Mexico is the accessibility of migration networks (Menjivar, 1995). Massey et al. (1993) define social migration networks as “sets of interpersonal ties that connect migrants, former migrants, and nonmigrants in origin and destination areas through ties of kinship, friendship, and shared community origin” (p. 447). Over the past 100 years Guanajuato has consistently been one of the highest migrant sending states in all Mexico, in large part due to the strong network ties and established migration networks (Massey, Durand, & Malone, 2002). Youth living in high migratory states such as Guanajuato are heavily influenced by the cultural expectation that they will migrate, and as many as 40% of them report an interest in working in the US, with around 35% reporting an interest in living there (Kandel & Massey, 2002). Unfortunately, the expectations to migrate are so high in some communities that youth who do not migrate are perceived as lazy or un-enterprising (Kandel & Massey, 2002). While the presence of community pressure surrounding migration expectations is well documented, its impact on adolescent quality of life and mental health has been understudied.

### 1.3 Suicide and Migration in Guanajuato

Researchers have found that suicidal ideation is associated with mobility, migration, and acculturation (Cho, & Haslam, 2010; Fortuna et al., 2007; Hovey, 2000; Ponizovsky, Ritsner, & Modai, 1999; Potter et al., 2001). Of these relationships, the connection between acculturation and suicidal ideation has received the most attention in the research literature. Unfortunately, very few resources have been invested in developing countries to better understand factors associated with suicide (Roth et al., 2011), and research looking at the association between suicidal ideation and factors *preceding* migration has received very little attention (Helliwell, 2007). Due to historically high migration rates, a rapidly increasing suicide rate, and a high percentage of youth suicides, the state of Guanajuato presents a unique opportunity to study the pre-migration connection between suicide and migration aspirations. In line with the assumption that suicidal ideation may be influenced by factors *preceding* migration (e.g., negative perception within the community), it is hypothesized that increased levels of suicidal ideation among youth in Guanajuato are associated with increased migration aspirations.

## 2. Method

This study is a secondary data analysis, and was approved by the Arizona State University Office of Research Integrity and Assurance [0610001162]. The original data were collected in 2007 as part of a comprehensive health survey among students enrolled in an alternative high school program located in Guanajuato, Mexico. The Videobachillerato (VIBA) alternative schooling program uses a combination of face-to-face and video education to reach students from traditionally low income families living in primarily rural areas of the state. At the time of data collection, there were 252 school centers in 38 municipalities with more than 25,000 enrolled students. Eight centers were randomly selected from 137 centers located in 20 municipalities within a 100 km radius of León, Guanajuato. Surveys were administered by the teachers in each school center. Of the 702 students who participated in the study, 60% were female and 40% were male. Although participant ages ranged from 14-24 years, 90% of students were of traditional high school ages (14-18). Youth over the age of 17 and those with families were not included in the final sample. The decision to exclude these youth was made to ensure that the information gathered regarding migration aspirations was based on individual hopes and desires rather than family circumstances (e.g., having to leave a spouse). The final analytic sample consisted of 507 participants.

### 2.1 Variables

The dependent variable, migration aspirations, was created by combining three questions: “Would you like to live in the United States some day?”, “Would you like to work in the United States some day?”, and “I am thinking about migrating to the United States some day.” Response options for the first two questions range from 0=“Not at all,” to 3=“Yes, a lot.” The third question’s response options range from 0=“Definitely not,” to 3=“Definitely.” When scaled together, the measure was found to have good internal reliability (α =.788).

The independent variable of interest was suicidal ideation. It was created by combining four questions: “During the past week, how frequently did you: 1) feel that you could not go on, 2) have thoughts about death, 3) feel that your family would be better off without you, 4) think about killing yourself.” There were 4 response options for each question: Not once, 1-2 days, 3-4 days, 5-7 days. The scale was also reliable among our sample population (α = .777). This same scale has been used in previous studies conducted in Mexico (González-Forteza et al., 1998).

Other variables included in this study were gender, age, socioeconomic status (SES), parent-child relationship, whether or not the respondent has ever been to the US, whether or not the respondent’s relatives have ever been to the US, and place of birth. Gender was included in the model because researchers have shown that Mexican boys and girls have different social norms and receive different messages about migration from their parents and the community (Kandel & Massey, 2002). Age was included because thoughts and plans regarding migration may be substantially different between a 14 year old respondent who just started high school and a 17 year old senior. SES was included because it has consistently been found to be a significant predictor of migration aspirations in previous research (Alba, Massey, & Rumbaut, 1999; Martin & Widgren, 2002). The SES measure in this study was a scale created from 5 question: “In your home, is there enough money to: 1) Buy food, 2) buy gasoline for your car or truck, 3) pay for basic services (light, water, etc), 4) buy clothing you need, 5) do fun things (take vacations, go to the movies, go out).” The responses categories were 0=Never, 1=Sometimes, 2=Almost always, and 3=Always. The internal reliability of this scale was strong (α=.854). A parent/child relationship variable was included in the models because the quality of that relationship can influence a youth’s desire or thoughts about leaving home. The measure was created from two questions: “How would you describe your relationship with your mother,” and “how would you describe your relationship with your father.” Responses ranged from 0= “Bad,” to 4=“Excellent.” The mean of both responses was calculated for each child; or, if they only had one parent, the score from the one parent was used. The measure addressing place of birth was included because adolescents who grow up in Guanajuato and are exposed to the societal expectations of international migration will likely have different feelings about migration when compared to youth who grow up in communities where migration is less important. This variable asked: "Where were you born?" Response options were dichotomized into two categories: 0) “Born in Guanajuato,” and 1) “Not born in Guanajuato.” Finally, two questions asking whether or not the respondent or a family member had been to the US were included in the model. Having been to the US previously, or knowing a family member who had, can influence youth migration aspirations – especially in Guanajuato communities that have long histories of circular migration (Massey, Durand, & Malone, 2002).

### 2.2 Analysis Strategy

First, descriptive statistics and bivariate analyses were run using SPSS 18 to identify correlations between measures. Then, STATA 11 was used to run linear regression models. Due to anticipated gender differences, the first model included only female participants, followed by a second model which was composed of only male respondents. After the gendered models were run, a final model was run with all participants. Additional statistical tests were used to see if gender moderated the relationship between suicidal ideation and migration aspirations.

## 3. Results

Table 1 shows descriptive statistics for all variables. The average participant age was 16.04 years (*SD* = 0.78), and 65% of the sample was female. While only 7% of students reported ever having been to the US (*M* = 0.07, *SD* = 0.25), the average participant had more than 3 relatives who had been to the US (*M* = 3.42, *SD* = 1.79). Over 95% of the participants were born in Guanajuato.

**Table 1:** Descriptive statistics

Variable	*N*	Range	Mean	Standard Deviation
Intentions to migrate	486	0-3	1.60	0.86
Suicidal thoughts during previous week	493	0-3	0.42	0.58
Gender (Fem=0)	500	0-1	0.35	0.48
Age	507	14-17	16.04	0.78
SES	506	0-3	1.90	0.57
Parent Child Relationship	495	0-4	2.93	0.90
Ever been to the US	466	0-1	0.07	0.25
# of relatives ever been to the US	500	0-9	3.42	1.79
Born in Guanajuato	504	0-1	0.04	0.20

Table 2 shows the OLS regression results predicting migration aspirations. The primary variable of interest, suicidal ideation, was a statistically significant predictor of higher migration aspirations among girls (*b* = 0.16; *p* < 0.05), for the entire sample (*b* = 0.18; *p* < 0.01), and was moderately significant for boys (*b* = 0.28; *p* < 0.10). Gender was a statistically significant predictor of migration aspirations when included in the 3^rd^ model (*b* = 0.40; *p* < 0.001), showing that males reported higher migration aspirations than females; however, the interaction models testing to see if gender moderated the relationship between suicidal ideation and migration aspirations were not significant (interaction models not shown). Older females reported higher levels of migration aspirations than younger females (*b* = 0.12; *p* < 0.05), but age was not a significant factor in either the male-only or full-sample models. Higher SES was a moderately significant predictor of migration aspirations among the female sub-sample (*b* = 0.15; *p* < 0.10), but was not significant in either of the other models. Likewise, in the female only sample, girls reporting lower parent-child relationship scores were more likely to have migration aspirations (*b* = -0.14; *p* < 0.05), while this variable was not significant in the other models. While having ever been to the US was only a moderately significant predictor of migration aspirations in the female-only model (*b* = 0.23; *p* < 0.10), having more relatives who had been to the US significantly predicted migration aspirations in all models (Model 1, *b* = 0.14; *p* < 0.001; Model 2, *b* = 0.12; *p* < 0.001; Model 3, *b* = 0.13; *p* < 0.001). Being born in Guanajuato was a statistically significant predictor of increased migration aspirations in the final model (*b* = -0.43; *p* < 0.05).

**Table 2:** OLS regression results predicting migration intentions

Independent Variables	Females	Males	All
Suicidal thoughts during previous week	0.16*	0.28†	0.18**
Gender (Fem=0 Mal=1)			0.40***
Age	0.12*	-0.02	0.07
SES	0.15†	0.04	0.10
Parent Child Relationship	-0.14*	0.05	-0.08
Ever been to the US	0.23†	0.12	0.20
# of relatives ever been to the US	0.14***	0.12***	0.13***
Born in Guanajuato	0.37	0.49	0.43*
			
R^2^	0.14	0.12	0.15

† p<.10;

* p<.05;

** p<.01;

*** p<.001

## 4. Discussion

The purpose of this study was to identify the connection between suicidal ideation and migration aspirations among youth in central Mexico. In line with the hypothesized connection, the results show that higher levels of recent suicidal ideation are predictive of migration aspirations. While previous researchers have explained the connection between suicidal ideation and migration using the healthy migrant theory (e.g., Shai & Rosenwaike, 1987; Sorlie et al., 1993) and acculturation-related mental health research (e.g., Hovey, 2000; Ponizovsky, Ritsner & Modai, 1999; Potter et al., 2001), these results suggest that factors such as acculturation stress may not be *causing* suicidal ideation among new migrants, but may instead be producing a resurfacing or worsening of previous mental health issues. Perhaps in the same manner in which large-scale violence (Martin & Widgren, 2002) and domestic violence (Klevens, 2007) are associated with migration, it is possible that suicidal ideation is a burden that instigates the desire to leave one’s current situation. The presence of a significant association between suicidal ideation and migration aspirations among youth living in Mexico adds a new perspective to the ongoing discussions surrounding migration and mental health. While much of the research literature has been centered around the impact of migration on mental health, looking at the impact of mental health on migration paves the way for a more complete understanding of migrant mental health. As future studies delve into the complexities of pre-migration mental health among immigrants, our current understanding of post-migration mental health, acculturation, and assimilation will be strengthened.

As expected, gender differences were identified among the youth in our sample. Predictors of increased migration aspirations for females included being older, having previously been to the US, coming from a family with more financial resources, and having a poor relationship with their parents. For boys, however, none of the aforementioned variables were statistically significant predictors of their migration aspirations. These gendered results may be due to the unique culture in which these adolescents live. Boys are strongly encouraged and expected to migrate (Kandel & Massey, 2002), but girls are not. Since girls do not face societal pressure to migrate, factors such as age, SES, parent-child relationship, and previous time in the US may have a greater influence on their migration aspirations.

### 4.1 Limitations

There are limitations to this study that are important to note. First, as this is a secondary data analysis of a cross-sectional survey, no cause and effect statements can be made regarding suicidal ideation and migration. Future researchers should design longitudinal studies so they are able to make more definitive statements on this topic. Second, the dataset did not have all the desired variables. Questions about previous suicide attempts, suicidal ideation history, and other mental health issues could have provided important information and led to a more thorough understanding of the relationship between suicidal ideation and migration. Researchers should include these variables in future studies on this topic. Third, the migration aspirations scale has not undergone a thorough psychometric assessment. Although it was found to be adequately reliable for use among this population (α =.788), a more thorough assessment of its psychometrics should be conducted. Finally, the suicidal ideation measure was not validated for use with our specific population, although it too was found to be sufficiently reliable (α =.777). Despite these limitations, this study makes a significant contribution to our understanding of the migration/suicide relationship.

### 4.2 Implications for Practice, Policy and Research

The findings from this study have potential implications for social workers, policy makers, and researchers. Social workers who serve Mexican migrants living in the US should consider conducting historical assessments of suicidal ideation in order to best understand the source of the client’s mental health problems. Social workers in Guanajuato and other high migratory communities may consider developing methods of creating awareness of the association between adolescent suicidal ideation and migration, specifically as it relates to the potential problem of the community migration expectations. As cultural norms in high migratory communities place great value on adolescent male migration, such awareness efforts should be directed to all members of the community. For policy makers in both the US and Mexico, these findings suggest a need to further investigate the relationship between suicidal ideation and migration. Funding opportunities should be created to provide researchers with the ability to devise longitudinal studies looking at migration and mental health. Researchers should carefully design these studies so they are able to eliminate time ordering problems and draw causal inferences. Additional measures - particularly validated measures of suicide, depression, and migration aspirations - should be included in such studies to ensure the accuracy of findings. This type of research would provide a unique understanding of the connection between suicidal ideation and migration, and could offer insights on how to reduce suicidal thinking among both migrants and those with migration aspirations.
